# *Cheetah*: software for high-throughput reduction and analysis of serial femtosecond X-ray diffraction data

**DOI:** 10.1107/S1600576714007626

**Published:** 2014-05-29

**Authors:** Anton Barty, Richard A. Kirian, Filipe R. N. C. Maia, Max Hantke, Chun Hong Yoon, Thomas A. White, Henry Chapman

**Affiliations:** a Centre for Free Electron Laser Science, Deutsches Elektronen Synchrotron DESY, Notkestrasse 85, Hamburg 22607, Germany; b Laboratory of Molecular Biophysics, Department of Cell and Molecular Biology, Uppsala University, Uppsala, Sweden; c NERSC, Lawrence Berkeley National Laboratory, Berkeley, California, USA; d European XFEL GmbH, Albert Einstein Ring 19, Hamburg 22761, Germany; e University of Hamburg, Luruper Chaussee 14, Hamburg 22761, Germany

**Keywords:** free-electron lasers, serial crystallography, serial X-ray diffraction

## Abstract

The emerging technique of serial X-ray diffraction, in which diffraction data are collected from samples flowing across a pulsed X-ray source at repetition rates of 100 Hz or higher, has necessitated the development of new software in order to handle the large data volumes produced. Sorting of data according to different criteria and rapid filtering of events to retain only diffraction patterns of interest results in significant reductions in data volume, thereby simplifying subsequent data analysis and management tasks. Meanwhile the generation of reduced data in the form of virtual powder patterns, radial stacks, histograms and other meta data creates data set summaries for analysis and overall experiment evaluation. Rapid data reduction early in the analysis pipeline is proving to be an essential first step in serial imaging experiments, prompting the authors to make the tool described in this article available to the general community. Originally developed for experiments at X-ray free-electron lasers, the software is based on a modular facility-independent library to promote portability between different experiments and is available under version 3 or later of the GNU General Public License.

## Abstract 2

The emerging technique of serial X-ray diffraction requires new software tools for the efficient analysis of large volumes of data. Event selection early in the analysis pipeline is highly advantageous. The described software for classifying, sorting and analysing events is freely available to the general community.

## 1. Introduction   

Serial X-ray diffraction using X-ray free-electron laser (XFEL) sources, in particular the expanding technique of serial femtosecond crystallography (SFX), is revolutionizing biological structure determination. Using X-ray pulses that outrun the effects of radiation damage, the X-ray dose can be more than a thousand times higher than that achievable with conventional X-ray sources, enabling measurements from crystals at room temperature, crystals that are too small for easy study at synchrotron sources or where time resolution is desired to trace the path of biochemical reactions (Chapman *et al.*, 2011 ▶; Seibert *et al.*, 2011 ▶; Redecke *et al.*, 2013 ▶). Such experiments generate large quantities of data that must be rapidly processed and analysed. The Coherent X-ray Imaging instrument at the Linac Coherent Light Source (LCLS), for example, delivers full frames of data at up to 120 Hz, resulting in 432 000 diffraction patterns per hour and data sets of tens to hundreds of terabytes in size. Analysing such large data sets is challenging, especially for small research groups, necessitating the development of new ‘big data’ paradigms in X-ray diffraction data processing.

To address the pressing issue of processing big data sets in serial X-ray diffraction we have developed a set of data analysis tools for serial imaging, specifically designed with the task of processing large data sets in mind. This software, called *Cheetah*, evaluates key data quality metrics such as number of Bragg peaks or maximum resolution, retaining only frames with a high likelihood of being usable for structure determination or further analysis from the stream of millions of detector frames, whilst producing condensed data such as virtual powder patterns, radial intensity profiles and peak lists for subsequent analysis. Reduced data are output in a facility-independent HDF5 output, including the CXI (Maia, 2012 ▶) data format, enabling downstream analysis programs such as *CrystFEL* (White *et al.*, 2012 ▶) to be written in an instrument- and facility-independent manner. This is particularly important given the plethora of unique file formats and interfaces developing at each experimental facility. The core functions of *Cheetah* are implemented as a plain C++ library for portability between facility-dependent file formats and to maximize the potential for code reuse.

The purpose of *Cheetah* is to evaluate the quality of each data frame for rapid feedback on experiment progress, reject data frames that should not be subjected to further analysis and perform the data pre-processing steps that are required for subsequent analysis. Data can be sorted according to various criteria and compiled into reduced forms such as virtual powder and radial stacks, as defined later in this paper, for subsequent analysis. To this end *Cheetah* performs the following primary functions:

(1) Correction of detector artefacts not already handled at the readout stage

(2) Estimation and subtraction of photon background

(3) Hit finding and frame sorting

(4) Identification and integration of Bragg peaks

(5) Generation of virtual powder diffraction patterns and radial lineout stacks

(6) Generation of statistics on hit rate and resolution

(7) Conversion of selected frames into a facility-independent format for subsequent analysis

Data are output in HDF5 format, providing instrument-, background- and geometry-corrected data in a portable structure reusable across multiple facilities.

## 2. Detailed description   

Key steps in the data analysis chain are described in detail below. The goal is to find a workable balance between computational efficiency and robustness of analysis, so that the many terabytes of data typically collected are efficiently reduced to a more manageable data volume worthy of more detailed analysis. Minimum user intervention is desired so that *Cheetah* can run autonomously and largely unsupervised either in real time or using batch processing on saved data. Rapid execution is desirable for fast feedback during the course of an experiment, ideally as close to real time as possible.

### 2.1. Detector corrections   

#### 2.1.1. Correction for detector artefacts   

*Cheetah* includes modules for the correction of detector artefacts: saturated pixels are identified and flagged, after which detector offsets determined from X-ray-free dark frames (dark calibration) are subtracted, followed by estimates of the common mode offset on each module (additive fluctuation in offset on individual modules). Pixels are corrected for individual gain variations (gain calibration) and finally known bad pixels are masked out. Nonlinearity in detector response can be rectified and detector-specific corrections applied. Routines are included for the generation of dark calibration data from X-ray-free data sets and gain calibrations from data sets with uniform detector illumination. These detector corrections are a standard part of any experimental analysis: they are included because the high data rates currently prevent these steps from being performed at the time of detector readout, and thus they must be performed as a part of the data analysis. Detector correction functions in *Cheetah* can be individually turned on or off as needed when detector correction functions are incorporated as a part of facility-provided analysis packages. User-defined masks can be loaded to separately define bad pixels and detector regions to be ignored during analysis.

#### 2.1.2. Geometry specification for segmented detectors   

The femtosecond-duration pulses delivered by X-ray free-electron lasers have necessitated the development of new detector technologies capable of integrating all photons arriving within the space of a few femtoseconds whilst sustaining full-frame readout at the FEL pulse repetition rate. Many of these detectors consist of multiple discrete detector modules tiled together to form one large detector. Specifying the location and relative position of pixels in a detector is critical to image analysis.

For example, the CSPAD detector (Hart *et al.*, 2012 ▶) used in the CXI instrument at LCLS consists of 64 separate modules, of 194 × 185 pixels each, tiled to produce a 2 megapixel detector of just over 1500 × 1500 pixels in size, read out at the LCLS repetition rate of 120 Hz. Individual modules or groups of modules may be moveable with respect to one another, and the pixel grid may not be perfectly aligned in translation or rotation between different modules. Meanwhile, the pnCCD detector can continuously save full frames at up to 200 Hz with 50 eV spectral resolution (Strüder *et al.*, 2010 ▶) using two independently moveable 512 × 1024 pixel modules located on either side of the direct beam. Detectors planned for use at the European XFEL will achieve a large detection area by tiling together many small high-speed detectors.

The following practical problems are encountered when analysing data from detectors composed of multiple modules:

(1) mechanical mounting of individual modules may not ensure relative alignment of pixel columns and rows between modules, especially if modules or groups of modules can be separately translated to suit the experiment;

(2) the location of module boundaries must be known because they define regions of non-existent signal between modules;

(3) data from the detector may be saved in a layout bearing no resemblance to the physical layout of pixels on the detector; and

(4) the exact location of modules might not be known at the time initial data processing is performed and may instead be refined during subsequent analysis, in which case assembling a physically correct image during initial processing is pointless.

**Figure 1:**
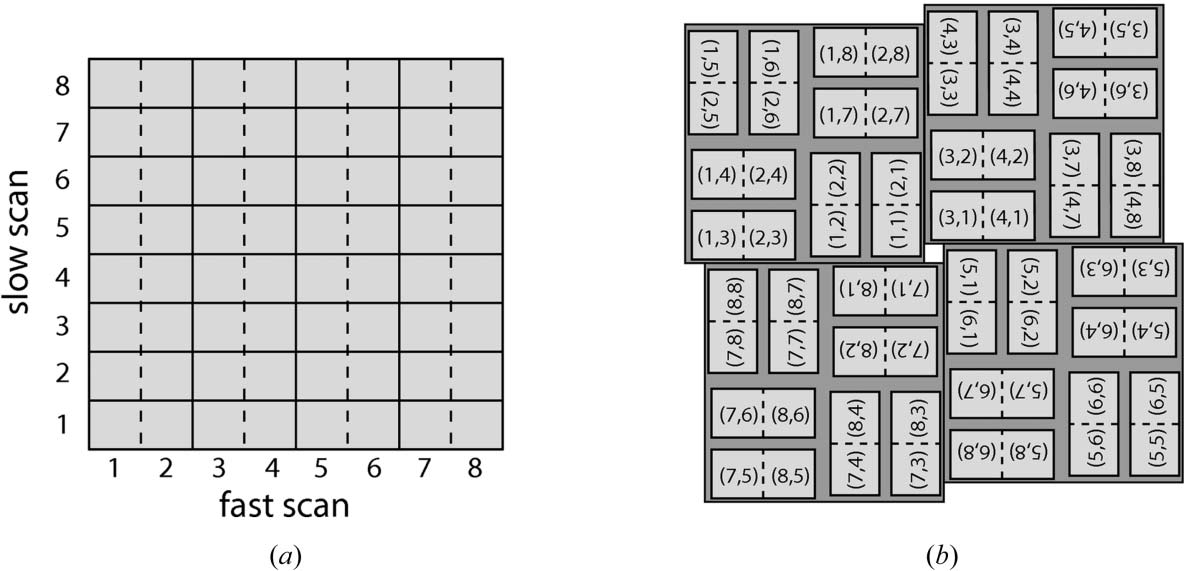
(*a*) ‘Raw’ non-interpolated layout of detector data (in this case for the CSPAD detector) with well defined module boundaries as internally represented for data processing. (*b*) ‘Assembled’ layout of the same modules as mounted on the physical detector system. A pixel map containing the coordinates of each data pixel in a suitably defined laboratory coordinate system is used to map between data in ‘raw’ layout and pixel locations in physical space.

We address these concerns by processing each module separately as an individual detector irrespective of its physical location or orientation. Analysis tasks such as background subtraction and peak finding are performed separately on each module to prevent artefacts caused by signal jumps at module boundaries. For example, the CSPAD detector data is arranged in memory into one non-interpolated two-dimensional array of 8 × 8 modules (1552 × 1480 pixels) in size with well defined module boundaries, as shown in Fig. 1 ▶(*a*). Once the locations of module boundaries are known, the majority of data reduction operations can be performed on data in this ‘raw’ layout.

**Figure 2:**
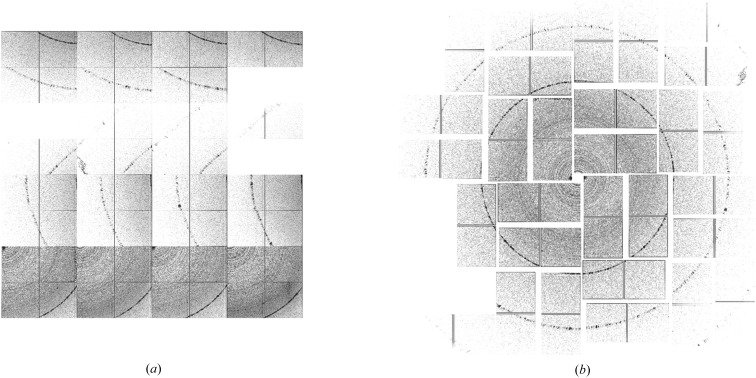
Virtual powder diffraction data (see §[Sec sec2.5.3]2.5.3) from many lysozyme nanocrystals in (*a*) the ‘raw’ non-interpolated layout of detector data and (*b*) the ‘assembled’ layout. Assembling a physically correct image requires interpolation of the raw data onto a regular pixel grid and results in irregular locations of individual module boundaries, due to the moveable central hole and mechanical tolerances in the placement of individual modules. The gaps between detector modules need to be accounted for in the analysis, and module geometry may be refined during subsequent analysis. For these reasons data analysis is performed in raw layout whenever possible.

Assembly of data into a physically correct layout, as shown in Fig. 1 ▶(*b*), is avoided if at all possible and only used for convenience when viewing images or when it is necessary to perform fast Fourier transforms (FFTs) of an entire diffraction image. This approach avoids interpolation errors when individual row and column pixels are not perfectly aligned between modules, and errors that arise because precise knowledge of module boundaries is typically discarded when individual modules are assembled into a single image prior to analysis. A virtual powder diffraction pattern formed by summing diffraction from many lysozyme nanocrystals in both raw and assembled layouts is shown in Fig. 2 ▶, illustrating clearly the different pixel layout between raw and assembled data.

Accurate specification of detector geometry is critical for certain types of analysis, for example radial integration of powder patterns or indexing of nanocrystal diffraction patterns. The detector geometry is specified in a pixel location map containing the coordinates of each detector pixel in a suitably defined coordinate system: this pixel map serves as the link between the indices of pixels in the data stream and the physical detector geometry and is referred to whenever knowledge of physical pixel or event locations is required. In practice the physical locations of individual detector modules are first determined using optical metrology data, obtained on a coordinate measuring machine, and subsequently refined using Debye–Scherrer rings from virtual powder diffraction patterns, then further refined using the results of auto-indexing of Bragg spots from a known sample.

Since modular detectors are, at the time of writing, still somewhat of a novelty in X-ray science we note the following practical consequences of analysing non-assembled data as performed in *Cheetah*:

(1) Viewing images by eye is easier when data are presented in a physically correct layout, and this is performed by image viewing software when needed.

(2) Bragg peaks are identified in raw layout and their coordinates converted to physical scattering vectors (*k_x_*, *k_y_*, *k_z_*) using the detector calibration pixel map for indexing and integration of reflections. There is no need to produce an interpolated image as the coordinates *k_x_*, *k_y_*, *k_z_* on the curved Ewald sphere can be calculated directly from detector coordinates for each pixel.

(3) For assembling data into three-dimensional diffraction volumes, coordinates on the Ewald sphere can be calculated directly from the pixel map for mapping into three-dimensional diffraction space – there is once again no real need to produce an interpolated image.

(4) Fast Fourier transforms need an array with regular pixel spacing and therefore require assembly of a physically correct image before Fourier transformation. Correction for Ewald sphere curvature can be performed at the same time as image assembly and may be required even if the detector has perfect pixel alignment.

(5) The main reason to perform image assembly is for interfacing with existing image analysis programs that assume a detector with regularly spaced pixels on a regular grid. For such programs, data must be interpolated onto a regular grid, or detectors must be constructed in such a way as to ensure sub-pixel accuracy in pixel alignment between modules. Such programs could in principle be rewritten for handle segmented detectors (for example, as performed in *CrystFEL*); however, extensive software rewriting may be neither practical, feasible nor even possible in many cases. *Cheetah* can output assembled data for use in such programs if needed.

#### 2.1.3. Pixel flagging   

Pixels are flagged during processing for special treatment as follows.

(1) A ‘bad pixel mask’ may be provided by the user (in the form of a binary image) to indicate pixels that have been identified as being unreliable: such pixels will not be considered for further analysis at any stage of data processing.

(2) A ‘peak mask’ may be provided to stipulate regions to be ignored specifically at the peak finding stage, which may be used to speed up the processing or to block regions of the detector that tend to produce erroneous peaks.

(3) Saturated pixels may be detected on a shot-by-shot basis by applying a simple user-specified intensity threshold (the saturation level is detector dependent).

(4) Regions of the detector that are physically shadowed from X-rays may be flagged, which is useful for determining background or electronic noise fluctuations.

(5) Unresponsive pixels that contain only an accurate measure of dark current and bias level may be flagged.

(6) A ‘resolution mask’, in the form of an annulus centred on the direct beam, may be generated from a user-specified detector resolution range; this is updated only when the detector is moved.

All of the above masks are optional and each affects only certain functions in subsequent analysis.

### 2.2. Subtraction of photon background   

Accurate subtraction of photon background is critical in diffraction image analysis – both for the analysis of Bragg peaks from crystalline samples and for the phasing of single-particle diffraction patterns. Practical experience indicates that most serial X-ray diffraction experiments have constantly changing background signals, owing to source fluctuations or to the sample itself; for samples flowing in a liquid or gas stream the femtosecond-duration X-ray pulses capture snapshot images of the background medium on time scales shorter than those of their intrinsic fluctuations. Background subtraction algorithms must take account of these fluctuations.

Background subtraction in *Cheetah* is performed in one of two ways. When the photon background is relatively steady from shot to shot but changes slowly over the course of many frames we subtract a background estimated from the recent history of non-hit frames. This ‘running background’ subtraction typically works well for samples in the gas phase and is relatively efficient to calculate; however, it can prove problematic for samples flowing in a liquid suspension where there is significant shot-to-shot variation in background. This is typically the case for serial nanocrystallography in a flowing liquid jet at resolutions where there are weak Bragg peaks mixed in with regions of relatively strong solvent scattering. For nanocrystals in liquid jets, local background subtraction on each pixel in each image is performed prior to determining Bragg peak locations; this is more computationally expensive but sometimes proves necessary for accurate Bragg peak characterization.

#### 2.2.1. Running background subtraction   

**Figure 3:**
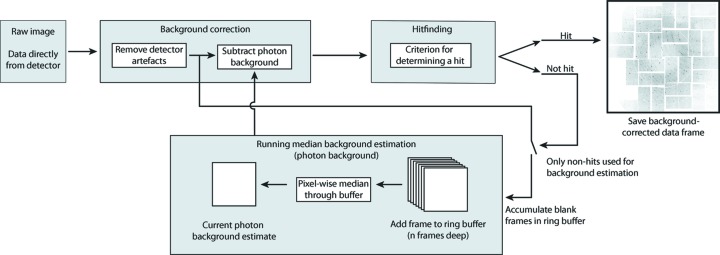
Frames identified as non-hits are added to a buffer of *n* images depth. A pixel-wise median through this buffer estimates the current photon background signal. Hot pixels and the standard deviation of background intensity are calculated from the same buffer.

Running background subtraction uses the many blank frames interleaved between hits to provide an up-to-date estimate of background signal in the data. Running background subtraction is performed by populating a buffer with the most recent non-hit frames and periodically calculating a pixel-wise median through this buffer, which is used to estimate the recent photon background (Fig. 3 ▶). Although a median filter is somewhat more computationally intensive than a simple average, we have found this median to provide a better background estimate than an average because simple averaging is more strongly affected by outlier pixel values. The buffer depth, *n*, is adjusted according to the rate of fluctuation in photon background, tempered by considerations of computational efficiency.

Running background subtraction works well when the photon background is relatively constant over the course of several data frames and provides a reasonable estimate of background from beamline optics, residual gas and any slow drifts in detector offsets. The background frame buffer serves multiple purposes, enabling hot pixels to be identified (pixels with abnormally high signal in more than 80% of frames in the buffer are excluded from subsequent analysis) and the standard deviation of the background to be calculated (used for determining signal-to-noise on a per-pixel basis). Additionally, static detector offsets are automatically incorporated into the running background, obviating the need for careful calibration of detector dark frames. Typically a buffer of *n* = 50 or *n* = 100 is used, which at the LCLS is about 0.5–1 s worth of data. Buffer depths of more than *n* = 500 typically provide no added benefit, because residual signal is affected more by shot-to-shot fluctuations than poor sampling of background frames.

#### 2.2.2. Local background subtraction   

**Figure 4:**
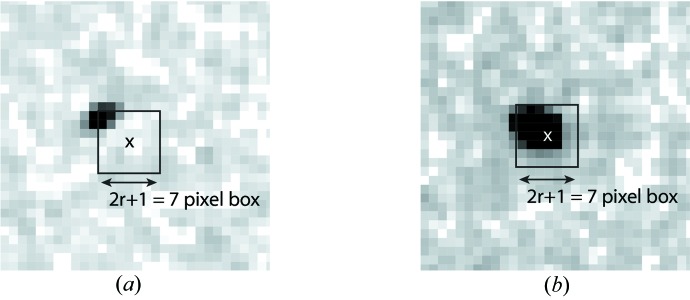
In the case of data from crystalline samples forming well defined Bragg peaks, the local background in the vicinity of a pixel is estimated as the median of pixel values in a box of side length 2*r* + 1 either side of the pixel of interest. For small peaks (*a*), the median of pixels within this box serves as a reasonable blind estimate of the background signal. However, when the peak becomes large compared to the box size (*b*), a simple median no longer serves to estimate the background alone. For our experiments to date, we have found that the number of pixels in the box should be at least three times the number of pixels in the peak.

For samples delivered in a liquid jet we observe that scattering from the liquid jet itself can vary significantly from shot to shot. Consequently a running background estimate may not always adequately account for this shot-to-shot variation in background signal and can be problematic for analysis. However, for crystalline samples (Boutet *et al.*, 2012 ▶; Redecke *et al.*, 2013 ▶), we know in advance that diffraction from the sample will form small and sharply defined Bragg peaks when compared to the photon background consisting of diffuse scatter and solvent scattering that vary on relatively long length scales. For such samples we find it effective to perform local background subtraction across the entire image: the background in the vicinity of a pixel is estimated as the median of all pixel values in a box of side length 2*r* + 1 either side of the pixel of interest (taking care to avoid running over the edge of detector module boundaries) (Fig. 4 ▶). Provided the box is sufficiently large compared to the size of the Bragg peaks, the majority of pixels will contain background signal rather than signal from the peak. The blind median of all pixel values within this box will serve as an adequate estimate of the local background for the purposes of peak detection and screening, provided the area of the box is at least twice the area of any potential Bragg peaks. We note that local background subtraction obviates the need to accurately specify a dark calibration as any static detector offsets are automatically accounted for during local background subtraction.

Selection of the correct background region is necessary to prevent excessive modification of Bragg peak intensities. For our experiments to date, we have found that the number of pixels in the local background region should be at least three times the number of pixels in the peak. For small and sharp, well separated peaks the local background region can be relatively small and thus the calculation relatively fast. As peak size increases the local background region must also be increased, slowing down the background calculation. On the other hand, a fast calculation may be preferable for screening purposes, provided peak finding is not adversely affected. A practical approach is to (1) apply a small local background radius to determine which frames have sufficient peak-like structures to be of interest, possibly using only a portion of the detector; (2) apply a larger local background radius to interesting frames only for determining the location of all peak-like structures in the frame; and (3) save image data with only detector artefact correction applied, along with identified peak locations for structure factor analysis. Modification of Bragg peak intensity is undesirable for integration of reflection intensities, making the saving of frames without local background subtraction advisable for subsequent analysis (the user can, of course, decide to do otherwise). In this way local background subtraction is used only for peak finding purposes, with original detector values preserved for subsequent analysis.

**Figure 5:**
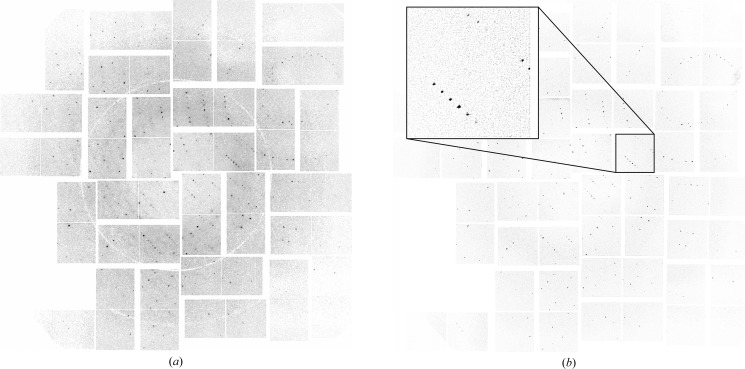
Comparison of results from running background subtraction and local background subtraction for crystalline samples flowing in a water jet. (*a*) Image after subtraction of a water ring averaged over multiple frames; fluctuations in pulse intensity and water jet structure result in imperfect background subtraction using running background subtraction. (*b*) Subtraction of local background using a moving median filter of width 7 pixels produces a cleaner image for peak detection.

A comparison of results from running background subtraction and local background subtraction for crystalline samples flowing in a water jet is shown in Fig. 5 ▶.

### 2.3. Image analysis   

#### 2.3.1. Identification of possible Bragg peaks   

Peaks in the intensity are identified as connected clusters of more than *n*_min_ pixels but fewer than *n*_max_ pixels in which every pixel has counts above a threshold value. The lower limit *n*_min_ serves to reject single-pixel outliers, whilst the upper limit *n*_max_ rejects overly diffuse peaks. The entire image is scanned, taking care to process each detector module separately and excluding any masked areas.

For most cases the following procedure serves to identify the majority of peaks in the image:

(1) Starting with the first pixel in memory, if the pixel intensity is below a specified static threshold [in raw analog-to-digital units (ADU)], move on to the next pixel.

(2) If the pixel intensity is above a specified signal threshold, locate all connected pixels that also meet the above criterion, whilst not crossing a detector module boundary. If the number of connected pixels falls within the range [*n*_min_, *n*_max_], then the connected region will be counted as a peak. Connected pixels are masked once evaluated to avoid the possibility of counting them twice in further analysis.

(3) The centroid of the peak and total intensity are calculated and used as the pixel location and intensity.

(4) Background noise around the peak is calculated, and the peak is retained only if the integrated intensity within the peak is sufficiently high above surrounding noise levels.

(5) Once a peak has been identified, move on to the next pixel and repeat until all pixels have been examined.

A constant threshold might suffice for samples with low background noise and sharp peaks. The most challenging samples, however, are ones that have weak peaks relative to the background. This background is often spatially varying, with notably elevated signal and noise in regions of solvent scattering. For such samples a static threshold no longer suffices. Instead, we exploit the largely radial symmetry of the noise profile to calculate a radially dependent threshold for the peak search. First the average intensity 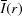 and standard deviation 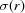 are calculated as a function of radial position *r* on the detector for each image immediately prior to the peak search. Obvious peaks are excluded from the SNR calculation using an iterative procedure. The peak search threshold is then given by the radially dependent value 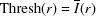
 + 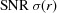, where SNR specifies the desired threshold in units of standard deviation within the annular shell. A minimum value of 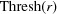 should be specified to avoid problems with almost empty frames. This algorithm is embodied in ‘peakfinder 8’. We have found values of SNR between 6 and 8 and a minimum threshold of 30 counts to be surprisingly robust for the analysis of data from the CXI instrument at LCLS across a range of samples and injection types. Values should be optimized to obtain the best results. 

When regions of the detector have significantly non-isotropic noise characteristics – for example, owing to coherent speckle in the water ring region – it may be hard to define one static threshold across the entire image. In such cases a second algorithm may be used, which applies a local intensity threshold based on local noise levels:

(1) Starting with the first pixel in memory, if the pixel intensity is below a specified threshold (in raw ADU), move on to the next pixel.

(2) If any of the eight nearest neighbour pixels have a greater intensity, move on to the next pixel.

(3) Once the brightest pixel in a region is found, calculate the mean background intensity 〈*I*〉 and standard deviation σ(*I*) within a concentric annulus of user-specified radius. The signal-to-noise ratio for this pixel is SNR = (*I* − 〈*I*〉)/σ(*I*).

(4) If either the background-corrected intensity (*I* − 〈*I*〉) or the SNR level of the pixel is below the user-defined thresholds, move on to the next pixel.

(5) The number of connected pixels that also meet the above criteria are counted. If the number of connected pixels falls within the range [*n*_min_, *n*_max_], then the connected region will be counted as a peak. Connected pixels are masked once evaluated to avoid the possibility of counting them twice in further analysis.

(6) The centroid of the peak is calculated and used as the pixel location value.

(7) A test is performed to check that there is not another peak nearby (within a specified distance); if nearby peaks are found, only the one with the highest SNR will be kept.

(8) Once a peak has been identified, move on to the next pixel and repeat until all pixels have been examined.

Each detector module is analysed separately to avoid complications in crossing from one module to another, and local background intensity and signal-to-noise σ(*I*) can be calculated during the local background subtraction step to avoid redundant calculation steps. Each peak ends up being characterized according to its position (*X*, *Y*), integrated intensity (*I*) and signal-to-noise ratio (SNR).

Peak searching can be applied first to selected portions of the detector (for example, the inner 1/4 of the detector) and only extended to the rest of the detector if sufficient peaks are found in the initial search region. This provides a speedup of almost 4 times because time-consuming steps such as background subtraction and peak finding are first conducted on only a portion of the detector, quickly eliminating blank frames. Pre-screening a region of interest can significantly reduce time spent on blank frames, provided an appropriate region of interest can be defined. 

### 2.4. Identifying sample hits   

Once the tasks of background correction and image evaluation have been performed, events identified as potentially useful hits are flagged and exported to a facility-independent HDF5 file for subsequent analysis. Event selection is based on relatively simple criteria depending on sample type.

#### 2.4.1. Crystalline samples producing Bragg peaks   

For crystalline samples we require a minimum number of Bragg peaks to have been identified in the diffraction pattern for it to be classified as a ‘hit’. After photon background subtraction, peaks are identified and characterized by their area and SNR to ensure they are sufficiently above local noise levels to be real peaks consistent with Bragg diffraction from the sample and not spurious fluctuations in local intensity. Individual peaks are counted, and only diffraction patterns with more than *n*_peaks_ peaks are retained for further processing. No attempt is made to separate single or multiple crystal hits during the hit finding stage. Identification of the number of crystal lattices is left to more sophisticated programs developed for protein crystallography; such information may be useful for a variety of programs capable of indexing multiple crystal lattices.

We find that for protein nanocrystals a criterion of *n*_peaks_ > 20 is required for autoindexing using *CrystFEL* (White *et al.*, 2012 ▶): this criterion arising from the number of peaks required to auto-index diffraction patterns using the Fourier approaches employed in that software suite (Powell, 1999 ▶). A minimum of two Bragg peaks would be theoretically required for indexing and diffraction pattern orientation with respect to the reciprocal lattice in certain cases, provided other indexing approaches such as template matching are applied. A further criterion based on resolution can be applied, such that only diffraction patterns with a resolution above some predetermined value are retained, where resolution is defined as the radius of the circle containing 80% of peaks (to reduce the effect of outlier peaks on the resolution estimate). The number and maximum resolution of these peaks are used as metrics for determining whether the diffraction pattern should be retained for further analysis.

The question naturally arises as to how effective the *Cheetah* hit finding approach is compared with simpler approaches. To this end we compared *Cheetah* with the crystal hit finding approach employed by *CASS* (Foucar *et al.*, 2012 ▶), as described in the recent paper of Barends *et al.* (2013 ▶). The *CASS* algorithm employed in that paper checked for the presence of pixels above 2000 ADU in the central region of the detector, with the presence of at least one such pixel triggering a hit (Barends *et al.*, 2013 ▶, supplemental material). Whilst this algorithm performed adequately for the lysozyme data analysed in that paper, for a different sample (G protein-coupled receptor microcrystals in lipid cubic phase medium) the *CASS* logic identified 178 940 potential hits from 1 584 452 data frames, of which 7772 could be indexed (4.3% indexing rate). The *Cheetah* logic identified 10 173 hits from the same 1 584 452 data frames, from which 8738 frames could be indexed (85.9% indexing rate). On lysozyme in a liquid jet we can achieve indexing rates of 60% or higher using *Cheetah* output, with most non-indexed frames appearing to contain multiple crystal hits. This compares to the approximately 30% indexing rate reported by Barends *et al.* (2013 ▶) for lysozyme in a liquid jet. The indexing rate is a good estimate of the false positive rate, as it is the indexed frames that are ultimately useful for crystallographic data analysis. The addition of multiple crystal indexing software to *CrystFEL* may enable the use of multiple crystal hits, most of which appear to fail during indexing and are currently counted as false positives. These results suggest that the *Cheetah* peak finding approach is capable of identifying useful data (and eliminating useless data) with higher accuracy than a simpler approach, even though no attempt is made at lattice identification during hit finding. Such results are of course sample and experiment dependent, so generalization to other samples and conditions must be made with care.

#### 2.4.2. Scattering from noncrystalline samples for single-particle imaging   

For the case of single-particle scattering there are no convenient intense and highly localized Bragg peaks to be counted. Instead, the presence of increased photon scattering on the detector and the spatial distribution of those photons must be used to determine whether or not a particle has been hit. In principle the detection of elevated scattering should be an easy task, and indeed applying a threshold to the sum of intensities from all pixels within a certain region of the detector is often sufficient to find the strongest particle hits. However, the strongest scattering events are not necessarily the most interesting ones. Theoretical calculations predict that frames with as few as 100 scattered photons may be useful for structural determination (Fung *et al.*, 2008 ▶; Elser, 2009 ▶; Philipp *et al.*, 2012 ▶), and in practice it is observed that the strongest scattering events often originate from droplets, clusters or contamination rather than the particles of interest, whilst the frames of interest are comparatively weak (Yoon *et al.*, 2011 ▶). Detecting weak yet potentially useful hits is much more challenging than detecting the strongest hits.

A simple method to identify hits involves using running background subtraction to remove static offsets from the detector or background gas scattering, and using the same frame buffer to calculate the typical noise in a given pixel, measured as the standard deviation of values for each pixel through the same buffer of background frames. Regions with elevated scattering are identified as pixels where the pixel value in a given frame is above a noise threshold for that pixel, and the number of pixels matching the threshold criteria is counted. Experience suggests that counting pixels forms a more reliable discriminator than total integrated image intensity alone, owing to a reduced sensitivity to single pixels with randomly high values. The appropriate threshold value can be determined from per-pixel histograms of detector counts at low photon count rates. A typical threshold of *I*(*x*, *y*) > 3σ(*x*, *y*) is applied, and the resolution of a diffraction pattern is estimated as the radius of the 80th percentile of pixels above the threshold. For detectors capable of single-photon counting, a threshold set just below the ADU value expected for a single photon results in the counting of photon-containing pixels.

For extremely weakly scattering samples such as single biomolecules probed using hard X-rays, it is difficult to distinguish from noise using total photon counts or photon-containing pixels alone (Elser, 2009 ▶; Philipp *et al.*, 2012 ▶). This is because of the small number of photons scattered from the sample compared to background sources, including detector noise and unavoidable scattered photons from the instrument. In such cases it is necessary to perform more sophisticated statistical analysis (Loh, 2012 ▶) to separate undesired scattering from scattering from the sample. *Cheetah* produces histograms of the distribution of measured counts in every individual pixel on the detector. This enables per-pixel detection calibration through the identification of histogram features corresponding to the arrival of individual or multiple photons for use in photon counting. Pixels with anomalous statistical behaviour as observed in the histogram can be identified and excluded from analysis. Calculation of the statistical distribution of detector counts in the presence of instrument noise and background scattering enables identification of hits by calculation of the log-likelihood probability that the current frame matches the known background statistics. In this case the histogram of background scattering forms prior information, and frames with a divergent log-likelihood metric are identified as statistical outliers potentially arising from particle hits (Loh, 2012 ▶). The field of single-particle imaging is not yet as mature as that of serial crystallography, and new approaches will no doubt be developed in the future. In particular, the statistical treatment of instrument background, identification of weak particle hits above instrument noise and background photon scattering, and the treatment of backgrounds in particle orientation and alignment is an area of active research.

### 2.5. Program output   

#### 2.5.1. Cleaned and filtered image data   

One of the primary functions of *Cheetah* is the identification and saving of data frames worthy of further analysis. To this end data frames classified as hits can be output in a facility-independent HDF5 format containing instrument-, background- and geometry-corrected data for subsequent analysis. The translation of data into a facility-independent format enables downstream analysis programs such as *CrystFEL* (White *et al.*, 2012 ▶) to be written in a facility- and instrument-independent manner for maximum portability. This is particularly important in the light of facility file formats being highly customized and only readable by facility-written software with limited portability. Frames identified as hits are listed in a plain text file, which can be used for later selection of individual data frames from the raw data stream if desired.

**Figure 6:**
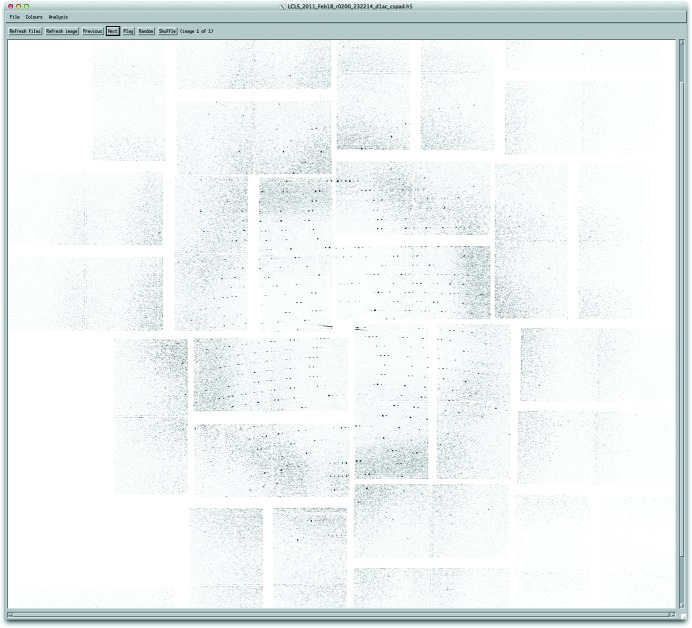
Cleaned and assembled image data of diffraction from a single-protein nanocrystal, as viewed in the viewer provided for reviewing *Cheetah* output.

Reviewing data frames selected as hits is one of the primary ways of monitoring the operation of *Cheetah*: to this end a simple image viewer (Fig. 6 ▶) is included for reviewing saved data frames and, for example, the accuracy of Bragg peak identification.

#### 2.5.2. Hit rate and spatial resolution   

**Figure 7:**
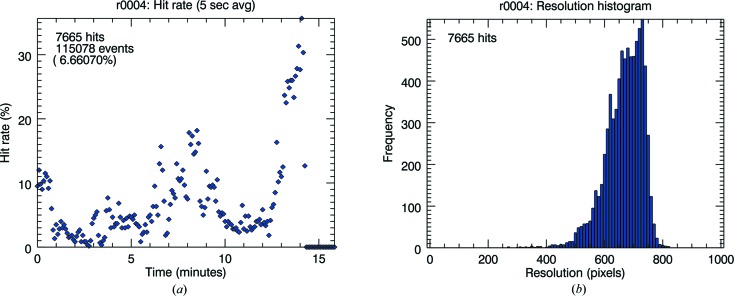
Statistics on hits identified in a given run in the form of hit rate (*a*) and distribution of resolution (*b*).

The calculation of overall hit rate and hit rate changes over time and is easy once events have been identified as either sample hits or blank frames. The overall hit rate is simply the total proportion of hits over the course of an experiment, whilst the instantaneous hit rate is the proportion of sample hits in the last *n* seconds, typically calculated as the percentage of hits averaged over 5 s intervals (5 s at the LCLS at 120 Hz pulse repetition rate amounts to 600 data frames). Time variations in hit rate are particularly useful in optimizing alignment during the experiment or diagnosing settling or clogging issues during sample delivery (Fig. 7 ▶). For each sample hit, diffraction is further classified according to total Bragg peak count (total number) and resolution (resolution of the circle containing 80% of Bragg peaks) to produce peak number and resolution histograms for individual data collection runs (Fig. 7 ▶). These statistics provide a useful summary of sample quality, alignment and achievable resolution for a given experiment.

Hit rates can very wildly depend on the experiment and sample delivery techniques. Nanocrystal diffraction in solution typically has hit rates of 10–15%, although extremes as low as 1% have been observed for dilute samples, whilst hit rates close to 100% have been observed for highly concentrated samples. Aerosol samples on the other hand typically produce 5–10% hit rates, although hit rates as high as 50% have been observed.

Low hit rates say more about the current state of the art in sample delivery technology than anything else. In an ideal experiment, each pulse would deliver a useful diffraction pattern. The percentage of rejected frames alone is thus a potentially misleading metric of hit finding ability, as it is also dependent on sample delivery strategies. The success of data weeding strategies should instead be measured according to the percentage of false negatives (useful hits which are rejected), the percentage of false positives (blank frames retained) and the particle discrimination rate [ability to accurately discriminate between useful sample (single particles or crystals) as opposed to junk (*e.g.* clusters, droplets)]. The currently available sample delivery techniques are far from achieving the goal of 100% useful data, and thus frame rejection strategies are currently very effective in reducing data volumes.

#### 2.5.3. Virtual powder patterns   

Traditional powder diffraction patterns are formed when many randomly oriented crystals are exposed simultaneously in the X-ray beam, producing characteristic Debye–Scherrer diffraction rings. On the other hand, in serial crystallography many individual crystals in random orientations are exposed to X-ray pulses one after another. Summing up multiple serial diffraction data frames therefore produces a diffraction pattern equivalent to powder diffraction from many crystals. We call this pattern a virtual powder diffraction pattern because it is formed by digital summation of many individual crystal diffraction patterns.

**Figure 8:**
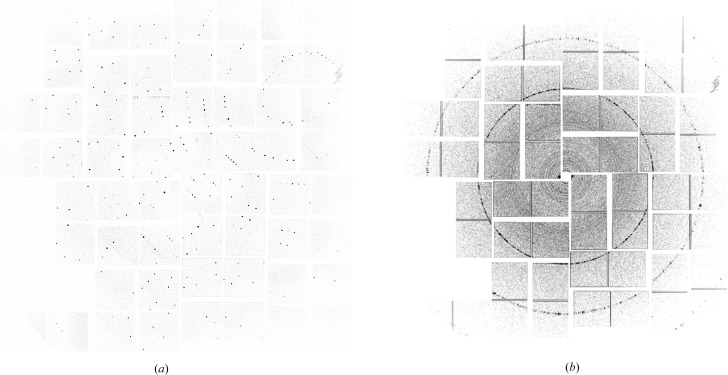
(*a*) One individual frame of background-corrected diffraction from a single lysozyme nanocrystal at the LCLS; and (*b*) the virtual powder diffraction pattern formed by summing of many thousand individual background-corrected frames.

One benefit provided by virtual powder diffraction is access to the diffraction patterns from individual crystals. Data frames without crystalline diffraction are excluded from the sum, reducing the contribution from solvent background, whilst background subtraction performed on each individual data frame enables only peaks, and not the background, to contribute to the virtual powder diffraction pattern. This results in a virtual powder pattern consisting of only the signal in Bragg peaks with greatly reduced background. Fig. 8 ▶(*a*) shows one individual frame of background-corrected diffraction from a single lysozyme nanocrystal at the LCLS, whilst Fig. 8 ▶(*b*) shows the virtual powder diffraction pattern formed by summing of many thousand individual background-corrected frames. Such virtual powder patterns are very useful for detector calibration and for quickly assessing the quality of data sets.

#### 2.5.4. Radial stacks   

**Figure 9:**
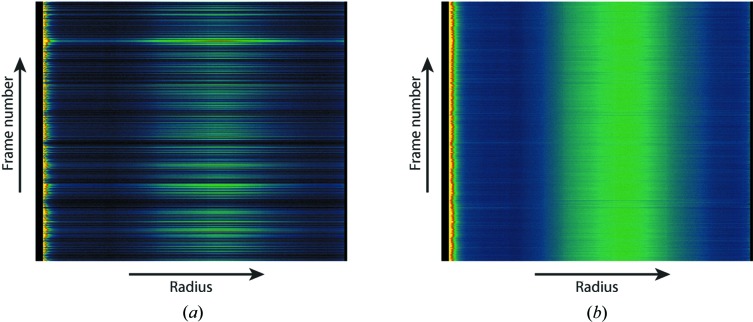
Radial stacks summarize the radially averaged signal for each frame (*a*) prior to normalization for shot-to-shot variation and (*b*) after normalization and outlier rejection. Radial stacks are used for WAXS/SAXS analysis and for comparing powder diffraction patterns on a shot-by-shot basis, and when sorted by laser delay or other reaction coordinates facilitate data evaluation in time-resolved studies.

Changes in the azimuthally integrated signal from successive frames can yield important information about the sample, for example in the case of time-resolved wide- and small-angle X-ray scattering analysis (WAXS/SAXS). Radial stacks where each row corresponds to the azimuthally integrated data from successive diffraction measurements are assembled for subsequent analysis, as shown in Fig. 9 ▶. The two-dimensional data in radial stacks significantly reduce data volume, compressing many gigabytes of full detector frames into a relatively small and manageable data set that can be easily analysed in separate programs or taken home for analysis. Stacks can be sorted on the basis of criteria such as sample excitation laser delay or laser-on and laser-off states, particularly when laser-on and laser-off states are interleaved. After outlier rejection and normalization for shot-by-shot variations in scattered intensity, radial stacks can be sorted according to reaction coordinate or crystal type in order to study dynamic evolution of states or other pheno­mena: different states with closely spaced or partially overlapping peaks in powder diffraction rings may be able to be sorted into different conformations through analysis of individual shot-by-shot powder diffraction patterns.

## 3. Implementation   

**Figure 10:**
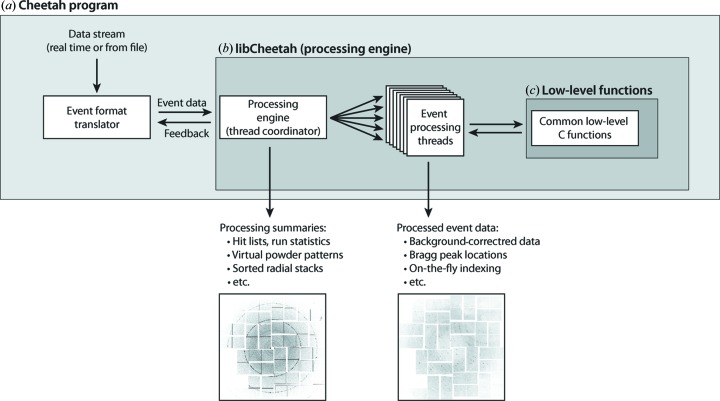
*Cheetah* implementation is multi-tiered. At the top level (*a*), *Cheetah* contains programs that interface to facility-dependent file formats and real-time data streams, translating and repackaging data from facility data formats for use by the *Cheetah* processing engine (*b*). Adaptation of this front end is all that is required to implement *Cheetah* with other facility data systems and file formats. The processing engine (*b*) is written in a facility-independent manner and compiled as a callable library, whilst core low-level functions (*c*) are implemented in plain C wherever possible to facilitate reuse of individual modules.

*Cheetah* is written in C++ and is available as source code under version 3 of the GNU General Public License (GPLv3). At the time of writing, the code can be downloaded from http://www.desy.de/~barty/cheetah/. Installation and usage instructions detailing the required libraries and computational environment are included. Data processing within *Cheetah* is multi-threaded: a single thread is responsible for reading data, with processing passed to multiple worker threads for independent processing (Fig. 10 ▶). Low-level functions are implemented in plain C wherever possible to facilitate reuse in other programs or use as callable functions in programs written in MATLAB (The MathWorks Inc., Natick, MA, USA) or Python.

*Cheetah* needs to interface with real-time data streams and data files in a variety of facilty-dependent formats. This complicates implementation because data formats and the programs capable of reading them differ from facility to facility. To address this problem we implemented *Cheetah* as a facility-independent library – libCheetah – that implements all necessary functionality in a framework written in portable C++ with minimal library dependencies. libCheetah compiles free from any dependence on facility-specific code and minimizes dependence on external libraries and packages as far as practically possible. Details of the library interface and documentation of low-level functions are found in the distribution.

The passing of experiment data to libCheetah functions is performed by a separate program which translates the data stream for use by libCheetah. The *Cheetah* front-end interface must be implemented within the data framework provided by the facility, or through another analysis program such as *CASS* (Foucar *et al.*, 2012 ▶). The provision of tools for reading custom data formats is necessarily a facility responsibility. *Cheetah* is implemented within the myana and psana framework at the LCLS, as well as for the SCALA HDF5 data format. Implementations for other frameworks and facilities will be incorporated into the *Cheetah* distribution as they are developed.

Separating tasks between a facility-dependent front end responsible for interfacing to the data stream and libCheetah which performs facility-independent analysis tasks simplifies debugging and enhances portability. Indeed, most problems compiling or executing *Cheetah* are observed to originate in this facility-dependent layer, including portability for reading data off-site and backwards compatibility of file formats over time.

Several scripts are included to assist in efficient execution of *Cheetah*. *hitfinder* is responsible for organizing the directory structure, ensuring that configuration files are copied into the correct locations, and executing *Cheetah* either in interactive mode or using a batch queue. *process* sets certain environment variables required by *hitfinder*, for example specifying the location of the *Cheetah* executable, the location of raw data and the destination for processed data, before executing *hitfinder. cheetahview* is provided for viewing output data frames and is especially useful for monitoring *hitfinder* output and ensuring that Bragg peaks are being accurately identified. *runstats* uses the *Cheetah* log files to compile statistics such as hit rate and resolution for individual data runs, while *powderplot* compiles one-dimensional radial averages from virtual powder patterns for WAXS analysis and enables the comparison of radially averaged virtual powder diffraction in one dimension between different runs.

## 4. Practical notes for users   

*Cheetah* can be obtained and installed from http://www.desy.de/~barty/cheetah/. Instructions for installation and use are included as a part of the package and will no doubt be more up-to-date than any instructions provided here. Once *Cheetah* is compiled and functional, the next step is to configure the scripts *process* and *hitfinder* for the current experiment and computational environment. These scripts specify the location of the *Cheetah* executable, necessary library paths and commands for batch queue submission, in addition to the location of raw data, configuration files, detector geometry data and the destination for processed data. Correctly configuring these housekeeping scripts greatly simplifies subsequent execution, enabling data to be processed with the single command process <run> <configuration>. Calibrations such as detector dark offset, per-pixel gain, and masks for known bad pixels and regions to be ignored can be specified, or are ignored if not present. The detector geometry should be specified using an HDF5 file containing the (*x*, *y*, *z*) location of each pixel on the detector as described in the documentation.

**Figure 11:**
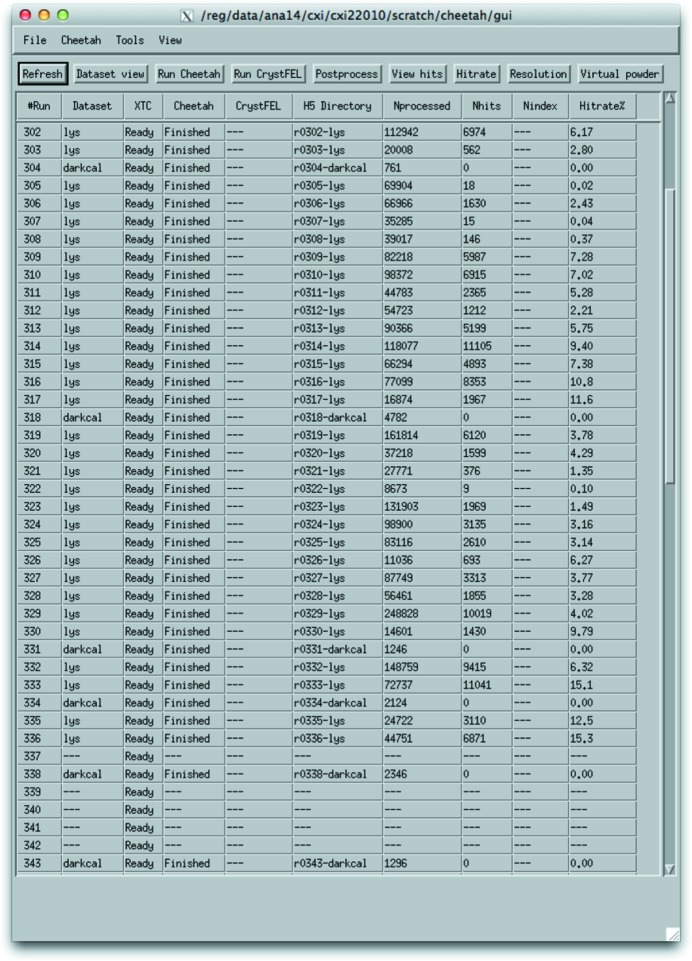
Control panel for *Cheetah* operation, showing currently available data sets, the progress of data processing and commonly used functions such as data reviewing.

Reviewing output is an important and necessary step in optimizing *hitfinder* output and background subtraction. Every experiment is different, even if of the same type as one conducted previously. Not optimizing the hit finding, *i.e.* expecting the default values to work, will most likely result in sub-optimal output and either too few hits or too many false positives. Reviewing output with an image viewer such as *cheetahview* or another program is essential. This program displays *Cheetah* output in either sequential order or a random order so you can get an unbiased sample of the output. For crystalline data, make sure that background is properly subtracted and Bragg peaks are accurately identified and circled, and that there are a minimum number of false Bragg peaks, while for noncrystalline data, check background subtraction, that areas of elevated scattering are accurately identified, and that the threshold area is neither too large nor too small for the given sample. A control panel is included for ease of use, showing data sets currently available for processing, confirming current processing status and providing quick access to commonly used functions (Fig. 11 ▶).

## 5. Future developments   

*Cheetah* is a new software project and is rapidly developing to keep pace with the emerging field of serial X-ray diffraction using FEL sources. Support for new facilities will be added as they come online, and new features are continually under development to meet the continually evolving needs of new experiments. One example is on-the-fly indexing and integration: as sample delivery methods improve, the proportion of useful data frames will increase and at some point translating data for each hit to a separate file format ceases to be efficient. *Cheetah* already identifies Bragg peak locations, integrates the signal above the background and is aware of detector geometry: this information could be passed directly to the *CrystFEL* library (White *et al.*, 2012 ▶) for auto-indexing on-the-fly without the need to save any intermediate data to disk. Another example for single-particle imaging is to incorporate morphological sorting and sizing so that only patterns matching the anticipated sample size and shape are retained for further analysis.

**Figure 12:**
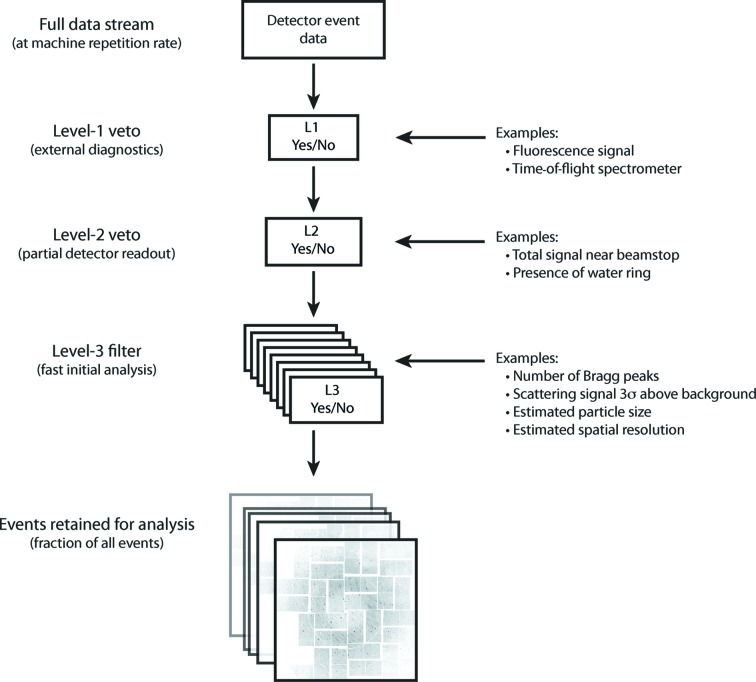
Event selection in serial X-ray diffraction experiments, borrowing from terminology used in particle physics experiments. Level-1 veto uses external diagnostics to determine whether a sample has been intersected by the X-ray pulse, while Level-2 vetoing relies on readout of only a portion of the detector. Level-3 event filters work in parallel, performing rapid analysis of the entire detector data to determine whether a particular event is worthy of retention for further analysis. *Cheetah* currently performs the role of a Level-3 event filter in addition to performing data reduction tasks.

Looking further ahead, future facilities such as the European XFEL will provide up to 27 000 pulses per second, representing a significant increase on the 120 Hz pulse repetition rate available today. At the European XFEL saving each and every frame for post analysis will no longer be practical and data reduction will ideally be performed in real time. Borrowing from terminology used in particle physics experiments (Bystricky *et al.*, 1997 ▶), the reduction of serial diffraction data may be described by three levels of event filter (Fig. 12 ▶). Level-1 triggers employ non-image-based diagnostics to determine whether or not the X-ray pulse hit a particle of interest. For example, fluorescence or time-of-flight ion spectroscopy may be able to quickly determine whether a given FEL pulse hit any sample and provide a veto signal prior to detector readout. Level-2 triggers use region-of-interest analysis to decide whether a frame should be discarded before all event data are read out, for example by integrating the total signal in a sub-region of the detector. The important point about Level-2 filters is that only small portions of the entire event data need be read out in order for a decision to be made, thereby reducing the total volume of data that must be read out from the instrument. Finally, Level 3 performs simplified science analysis on full event data to decide whether to discard the event or retain it for further analysis. Within this three-tier model, *Cheetah* currently performs the role of a Level-3 event filter. Certain portions of *Cheetah* may be suited to implementation at the L2 level, whilst research into alternative strategies for reliable L1 vetoing using external diagnostics is currently underway. Migrating as many functions as possible, such as hit finding or on-the-fly accumulation of radial stacks or autocorrelation functions, into field-programmable gate array hardware or intelligent pixels would further speed analysis and reduce overall data volumes. Development in this direction is critical for data volume management from serial diffraction experiments at the European XFEL.

## 6. Conclusions   

The use of online data rejection is essential in serial diffraction experiments. One recent structure determined at LCLS involved the collection of almost 4 million detector frames (20 TB) over 10 h of data collection; of these only 293 195 contained potential crystal diffraction patterns, of which only 178 875 (<1 TB) proved usable for structural analysis (Redecke *et al.*, 2013 ▶). To date the authors of this paper have processed over 1.2 PB of data using *Cheetah* from a total of 24 experiments at the LCLS, yet from this data only the relatively modest quantity of 80 TB of data frames have been extracted and used for detailed analysis. From such statistics it is evident that the reduction in data transfer, storage and downstream computation requirements resulting from rapid analysis and event selection can be significant, bringing such experiments within the reach of modest research groups who do not have access to large-scale computation or storage infrastructure. With experiments at LCLS producing half a million data frames per hour, the deployment of detectors capable of reading out hundreds of frames per second at synchrotron sources and frame rates of up to more than 90 million diffraction patterns per hour possible (27 000 frames per second) at the European XFEL, the move towards online data screening and rejection in serial diffraction is inevitable and represents a paradigm shift in X-ray data collection for a new generation of high-repetition-rate experiments.

*Cheetah* is free and open source software (GPLv3) and is available at http://www.desy.de/~barty/cheetah/.

## References

[bb1] Barends, T. R. M. *et al.* (2013). *Nature*, **505**, 244–247.

[bb2] Boutet, S. *et al.* (2012). *Science*, **337**, 362–364.

[bb3] Bystricky, J., Calvet, D., Ernwein, J., Gachelin, O., Hansl-Kozanecka, T., Hubbard, J. R., Huet, M., Le Du, P., Mandjavidze, I. & Mur, M. (1997). *IEEE Trans. Nucl. Sci.* **44**, 342–347.

[bb4] Chapman, H. N. *et al.* (2011). *Nature*, **470**, 73–77. 10.1038/nature09750 PMC3429598 21293373

[bb5] Elser, V. (2009). *IEEE Trans. Inf. Theory*, **55**, 4715–4722.

[bb6] Foucar, L. *et al.* (2012). *Comput. Phys. Commun.* **183**, 2207–2213.

[bb7] Fung, R., Shneerson, V., Saldin, D. K. & Ourmazd, A. (2008). *Nat. Phys.* **5**, 64–67.

[bb8] Hart, P. *et al.* (2012). *Proc. SPIE*, **8504**, 85040C.

[bb9] Loh, N. D. (2012). *Proc. SPIE*, **8500**, 85000K.

[bb10] Maia, F. R. (2012). *Nat. Methods*, **9**, 854–855. 10.1038/nmeth.2110 22936162

[bb11] Philipp, H. T., Ayyer, K., Tate, M. W., Elser, V. & Gruner, S. M. (2012). *Opt. Express*, **20**, 13129–13137. 10.1364/OE.20.013129 PMC3635695 22714341

[bb12] Powell, H. R. (1999). *Acta Cryst.* D**55**, 1690–1695. 10.1107/s0907444999009506 10531518

[bb14] Redecke, L. (2013). *Science*, **339**, 227–230.

[bb15] Seibert, M. M. *et al.* (2011). *Nature*, **470**, 78–81. 10.1038/nature09748 PMC4038304 21293374

[bb16] Strüder, L. *et al.* (2010). *Nucl. Instrum. Methods Phys. Res. Sect. A*, **614**, 483–496.

[bb17] White, T. A., Kirian, R. A., Martin, A. V., Aquila, A., Nass, K., Barty, A. & Chapman, H. N. (2012). *J. Appl. Cryst.* **45**, 335–341.

[bb18] Yoon, C. H. *et al.* (2011). *Opt. Express*, **19**, 16542–16549. 10.1364/OE.19.016542 21935018

